# Lesion-network mapping for tics: moving from replication to clinical translation

**DOI:** 10.1093/braincomms/fcad141

**Published:** 2023-04-28

**Authors:** Christos Ganos, Andreas Horn

**Affiliations:** Movement Disorder and Neuromodulation Unit, Department of Neurology, Charité – Universitätsmedizin Berlin, corporate member of Freie Universität Berlin and Humboldt- Universität zu Berlin, Department of Neurology, 10117 Berlin, Germany; Movement Disorder and Neuromodulation Unit, Department of Neurology, Charité – Universitätsmedizin Berlin, corporate member of Freie Universität Berlin and Humboldt- Universität zu Berlin, Department of Neurology, 10117 Berlin, Germany; Center for Brain Circuit Therapeutics, Department of Neurology Brigham & Women’s Hospital, Harvard Medical School, Boston, MA 02115, USA; MGH Neurosurgery & Center for Neurotechnology and Neurorecovery (CNTR) at MGH Neurology Massachusetts General Hospital, Harvard Medical School, Boston, MA 02114, USA

## Abstract

This scientific commentary refers to ‘Mapping a network for tics in Tourette syndrome using causal lesions and structural alterations’, by Zouki *et al*. (https://doi.org/10.1093/braincomms/fcad105).

**This scientific commentary refers to ‘Mapping a network for tics in Tourette syndrome using causal lesions and structural alterations’, by Zouki *et al*. (https://doi.org/10.1093/braincomms/fcad105).**

The study of tic pathophysiology has been in many ways difficult, not the least due to the heterogeneity of clinical presentations. These encompass differences in the characteristics of studied tic phenomena (e.g. from simple blinks to more complex repetitive behaviors; presence of premonitory urges; degrees of tic-awareness and voluntary tic control), as well as variability in their neuropsychiatric associations, which include attention-deficit hyperactivity disorder, obsessive-compulsive disorder, anxiety disorder, depression and autism spectrum disorder. Importantly, clinical profiles may change over the course of neural development further hampering the search for reliable pathophysiological insights across populations. Moreover, it is unclear whether some of the reported findings reflect the pathophysiological mechanisms of the repetitive motor behaviors, or their associated sensory experiences, or even mechanisms of tic control. In fact, studies in humans are often unable to disentangle brain changes that drive tic emergence from those that may be part of symptom compensation. This is also relevant for the study of the effects of behavioral or pharmacological interventions, as these may also induce wider brain changes well beyond a specific ‘tic network’. Although animal models attempt to abridge some of these issues, their external validity, including face validity (i.e. do jerky behaviors in rodents and primates correspond to tics or myoclonus?), is often challenged.

Lesion-network mapping has provided a new way to overcome this impasse in neuropsychiatric disorders, including tics, by offering causal insights into the structure of specific symptoms.^1^ We recently applied this method in tic disorders using 22 previously published cases, where the presence of new onset tic behaviors was causally attributed to a novel lesioning event.^2^ We demonstrated that tic-inducing lesions connected to a common network of areas that included insular cortices, cingulate gyrus, striatum and globus pallidus internus, as well as thalami and cerebellum. We demonstrate the same network to be relevant for deep brain stimulation (DBS) targeting, as was shown by Baldermann *et al*.,^3^ before. Using the same analytical approach but a partially different collection of cases, Zouki *et al*.^4^ now replicate our own previous findings. Specifically, they included 19 cases in their analysis, 11 of which overlapped with the ones we studied. However, they also included two cases, where the description of a facial ‘tic’ clearly referred to hemifacial spasm^5,6^ and a case where the clinical history and the accompanying video most likely demonstrate a functional neurological disorder.^7^ Zouki *et al*. further extended our previous findings by using additional analyses. Namely, one common concern in the study of lesion network mapping is that the pathophysiological blueprint of a specific clinical sign may differ in lesion-induced (secondary) syndromes than in the prototypical (primary) cases of a particular disorder, as for example in Tourette syndrome (TS). To address this, Zouki *et al*. selected some studies that examined brain morphometry in TS in children and adults and performed anatomical likelihood estimation (ALE) based meta-analysis to identify consistent differences in brain structure for the TS population compared to neurotypical controls. On a localized level, the ALE analysis did not show significant differences. However, when subjected to brain networks, a familiar result appeared. This was done using a technique that is closely related to lesion network mapping, termed coordinate network mapping.^8^ Indeed, the resulting map further confirmed the validity of the lesion-network and its pathophysiological relevance to *primary* tic disorders.

We should celebrate replication of results between three studies that showed involvement of the same network with tic disorders.^2-4^ This series of replications stands in contrast to the ongoing replication crisis in neuroimaging.^9^ A key reason for these successful replications might be that all studies applied strategies that leverage causal sources of information to study the brain, namely DBS network mapping by Baldermann *et al*.,^3^ DBS and lesion network mapping by Ganos *et al*.,^2^ and now lesion and coordinate network mapping by Zouki *et al*.^4^ Similar replications—which applied related techniques—have been already demonstrated for several other disorders, including depression^10,11^ and obsessive-compulsive disorder.^12^

Given these replicable effects, a key question is how to translate these findings back to clinical practice? One strategy could be to probe whether the tic network that emerged from the three studies has therapeutic potential as a neuromodulation target. In retrospective fashion, the studies by Baldermann *et al*.^3^ and Ganos *et al*.^2^ could already show that clinical outcomes of TS cases that either received neuromodulation in midline thalamic nuclei, or the anterior segment of the pallidum had stronger tic reductions the more the identified network had been modulated. A next step would be a prospective evaluation in carefully selected patients, specifically under the light that connectivity to additional brain areas (e.g. M1 and supplementary motor area) from other neurostimulation targets (e.g. the subthalamic nucleus) may also mediate symptom improvement.^13^ A second strategy would involve modulating the therapeutic tic network based on non-invasive neuromodulation, which may be applied either as a stand-alone intervention or to augment other treatments. Clearly, although these efforts would provide a coordinative blueprint to guide where neuromodulation should take effect, the exact stimulation type and protocols further require careful design, as the natural fluctuations of tic severity complicate the study on the effects of any intervention. Large-scale collaborations as the ones fostered within the Tic Disorders Study Group of the Movement Disorder Society, the International TS-DBS Public Database and Registry^14^ and as part of specific interest consortia^15^ currently ensure the rapid diffusion of information and enhance crosstalk between clinicians and researchers to collectively maximize our efforts on a global scale for the benefit of our patients.
